# Supplementary figures and images for: Insights into cork weathering regarding colour, chemical and cellular changes in view of outdoor applications
Source: PLoS One. 2024 Apr 4;19(4):e0301384. doi: 10.1371/journal.pone.0301384 (PMC10994410; doi:10.1371/journal.pone.0301384)

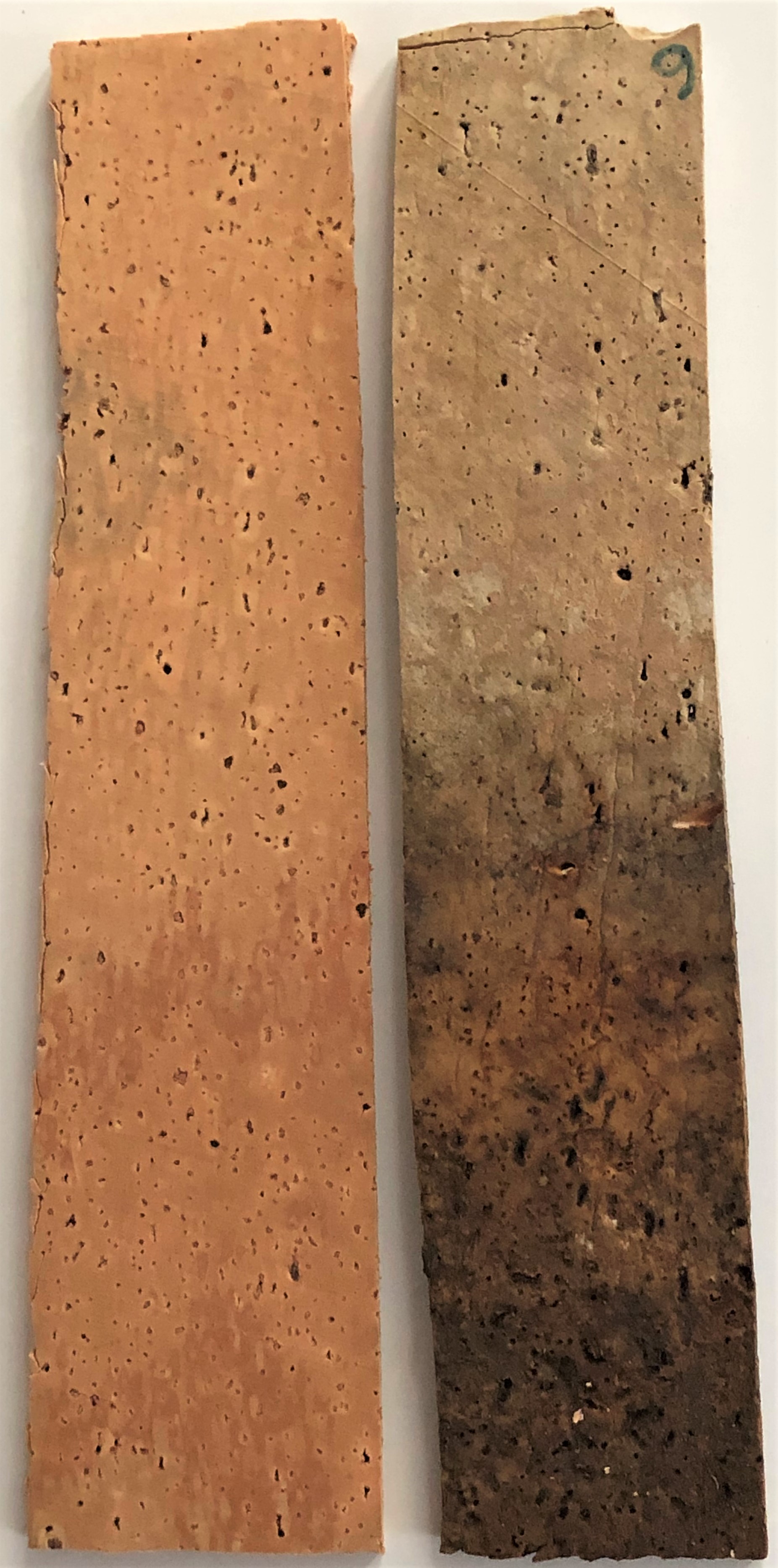

Supplement: S1 Fig — Photograph of one unexposed cork sample and of one 1-year exposed cork sample with outdoor exposure (right) and soil burial (left). (JPG) [file pone.0301384.s001.jpg]
