# Supplementary material for: Insights into cork weathering regarding colour, chemical and cellular changes in view of outdoor applications
Source: PLoS One. 2024 Apr 4;19(4):e0301384. doi: 10.1371/journal.pone.0301384 (PMC10994410; doi:10.1371/journal.pone.0301384)
Supplement: S2 Fig — (PDF) [file pone.0301384.s002.pdf]

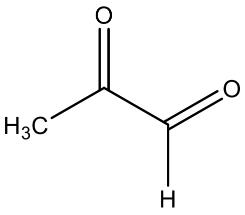

1) 2-oxo-propanal

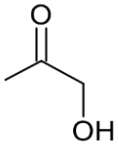

4) 1-hydroxy-2-propanone

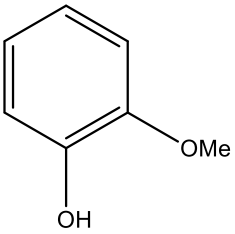

10) guaiacol

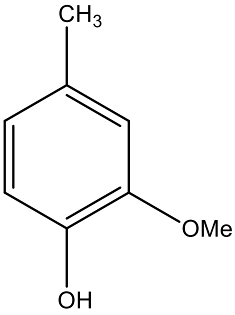

15) 4-methylguaiacol

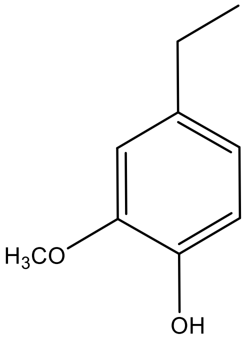

18) 4-ethylguaiacol

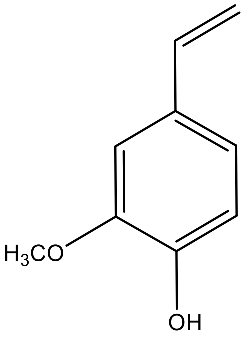

19) 4-vinylguaiacol

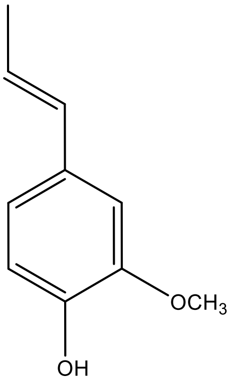

24) *trans* isoeugenol

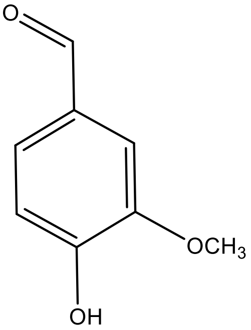

26) vanillin

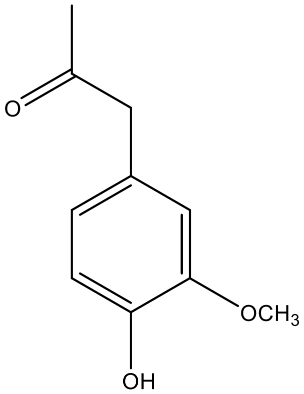

31) guaiacylacetone

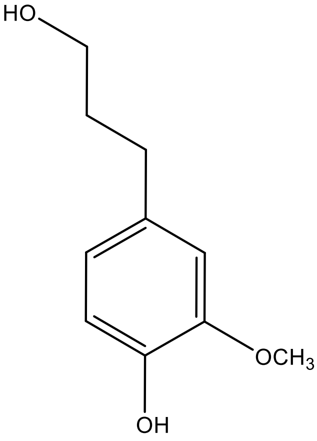

37) dihydroconiferyl alcohol

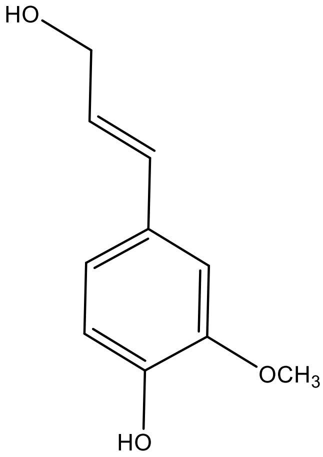

41) *trans* coniferyl alcohol

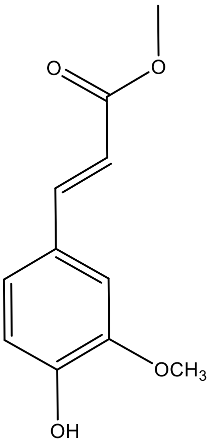

44) ferulic acid methyl ester
