# Supplementary material for: Insights into cork weathering regarding colour, chemical and cellular changes in view of outdoor applications
Source: PLoS One. 2024 Apr 4;19(4):e0301384. doi: 10.1371/journal.pone.0301384 (PMC10994410; doi:10.1371/journal.pone.0301384)
Supplement: Attachment — Submitted filename: Response to reviewers.docx [file pone.0301384.s003.docx]

**Response to reviewers**

Dear Editor,

The manuscript was revised following all the comments of the reviewers whom we warmly thank.

The response to each of the questions is given below.

The funder had no role in the study design, data collection and analysis, decision to publish, or preparation of the manuscript.

With our best regards

Helena Pereira , on behalf of the authors

**Comments to the Author**

1. Is the manuscript technically sound, and do the data support the conclusions?

The manuscript must describe a technically sound piece of scientific research with data that supports the conclusions. Experiments must have been conducted rigorously, with appropriate controls, replication, and sample sizes. The conclusions must be drawn appropriately based on the data presented.

Reviewer #1: Yes

Reviewer #2: Partly. Response: This was addressed in the revised manuscript, as described below

Reviewer #3: Yes2. Has the statistical analysis been performed appropriately and rigorously?

Reviewer #1: Yes

Reviewer #2: No: Response: This was addressed in the revised manuscript, as described below

Reviewer #3: Yes

3. Have the authors made all data underlying the findings in their manuscript fully available?
The [PLOS Data policy](http://www.plosone.org/static/policies.action#sharing) requires authors to make all data underlying the findings described in their manuscript fully available without restriction, with rare exception (please refer to the Data Availability Statement in the manuscript PDF file). The data should be provided as part of the manuscript or its supporting information, or deposited to a public repository. For example, in addition to summary statistics, the data points behind means, medians and variance measures should be available. If there are restrictions on publicly sharing data—e.g. participant privacy or use of data from a third party—those must be specified.

Reviewer #1: Yes

Reviewer #2: Yes

Reviewer #3: Yes

4. Is the manuscript presented in an intelligible fashion and written in standard English?

PLOS ONE does not copyedit accepted manuscripts, so the language in submitted articles must be clear, correct, and unambiguous. Any typographical or grammatical errors should be corrected at revision, so please note any specific errors here.

Reviewer #1: No : Response: This was addressed in the revised manuscript

Reviewer #2: No: Response: This was addressed in the revised manuscript

Reviewer #3: Yes

5. Review Comments to the Author

**Reviewer #1:**

Add the importance of study and relevance this study to international readers. Why this study should be done? what are the limitation of this study?

R.: Done. These aspects were added to the manuscript in the Introduction.

Double check the citation and the references list. Make sure all the citations were included in the references. The references should be adjusted as the journal guideline.

R.: Done. The references were checked and a few formatting errors wee corrected.

Check the grammatical errors.

R.: Done. The manuscript was checked for grammatical errors

In conclusion you should add the future research prospect related the present study.

R.: Done. This was added to the manuscript

**Reviewer #2**

In this manuscript, the authors investigate the effects of 1-year outdoor weathering and soil burial on the color and chemical composition of cork. This study found that except the color changes, the cork maintains its physical structure and integrity when applied in outdoor conditions, including both above and beneath the ground. This is an interesting study focusing on the not well-explored aspects of cork weathering. Their results provide evidence of the potential for using cork as a green material in building facades. However, in my opinion, this study lacks proper control and statistical analysis. Once these issues are addressed, I believe this paper will be a valuable addition to the PLOS ONE journal.

Major comments

Line 131: I suggest that the cork samples collected on day 0 would serve as a more appropriate control for comparisons of color and chemical composition after the 1-year experiment. Please provide additional details regarding the use of the side of the cork plank with less exposure to light as the reference.

R.: Clarification of the control used for measurements was made in more detail in the Experimental section of the manuscript. In fact we had a control for colour measurement for each sample before it was put in the field test, therefore the differences caused by the weathering along time were measured in the same sample.

2. I believe this study included the replications during their experiments, but I didn’t see the statistical analysis.

R.: Done. The statistical results were added to the manuscript for the colour measurements in M&M and in the Results.

3. Line 171: The cork samples were dried in an oven at 60 °C before microscopic and chemical analysis. Could this temperature be considered a harsh treatment for cork, potentially causing changes in color and chemical composition?

R.: This is a standard procedure of drying biomass materials, including cork. Cork is stable to temperatures quite above 60ºC and only starts to undergo small chemical changes by about 150ºC). In fact boiling cork with water at 100ºC is the common standard preparation step during industrial processing of cork.

Rosa, M. E. & Pereira, H. (1994). The effect of long term treatment at 100ºC - 150ºC on structure, chemical composition and compression behavior of cork. Holzforschung, 48(3): 226-232.

Minor comments:

1. Line 32: The letter "ç" in the word "façades" is not used in English. I believe this non-English character appears in all instances of "façade" throughout the manuscript.
R.: Façade is used in English (it is an appropriation from the French)

2. Lines 90 and 93: The full names of "CIELAB" and "FTIR-ART" should be provided when these terms first appear in the manuscript.

R.: Done.

3. Line 264: I recommend providing a photo of the cork samples after one year of exposure to demonstrate their physical integrity as the first figure.

R.: Done. A photograph was added to the manuscript as supplementary material (S1 Fig)

4. In figure 3 and 4, I recommend adding illustrations of the important chemical structures next to their corresponding peaks.

R.: Done. The most important chemical structures of Fig. 4 were added to the manuscript as supplementary material (S2 Fig),

5. Line 585: I believe the correct word should be “from” instead of “form”.

R.: Corrected

**Reviewer #3**

I recommend acceptance this manuscript as it is valuable and related to one of the most world challenges which is the pollution of the environment. As the author showed the different application of the cork and how it will be useful especially in the architecture and building field. In addition, cork showed a considerable structural and physical stability that allows its application in outdoor conditions.

R.: Thank you for your comments and appreciation
