# Supplementary material for: Insights into cork weathering regarding colour, chemical and cellular changes in view of outdoor applications
Source: PLoS One. 2024 Apr 4;19(4):e0301384. doi: 10.1371/journal.pone.0301384 (PMC10994410; doi:10.1371/journal.pone.0301384)
Supplement: Attachment — Submitted filename: Review 2nd round 030424.docx [file pone.0301384.s004.docx]

Upon careful examination of the revised manuscript and the authors' responses to the comments provided during the initial review, I believe the authors have adequately addressed all major concerns and most minor concerns. Regarding minor comment 1, my intention was not to suggest that the word “facade” is improper in English. Rather, I aimed to inquire whether the letter “ç” is commonly used in formal English writing. As the authors mentioned, this letter is indeed of French origin. I would suggest that the authors or the publication team at PLOS One verify its appropriateness in this context. Nonetheless, this is a minor issue. Once this matter is resolved, I believe the paper is ready for publication.
